# Rheumatoid arthritis and adverse pregnancy outcomes: a bidirectional two-sample mendelian randomization study

**DOI:** 10.1186/s12884-024-06698-3

**Published:** 2024-07-31

**Authors:** Tongmin Chang, Zengle Zhao, Xiaoyan Liu, Xuening Zhang, Yuan Zhang, Xinjie Liu, Yuan Zhang, Ming Lu

**Affiliations:** 1 School of Public Health, Cheeloo College of Medicine, Shandong University, Jinan, Shandong 250012 China; 2 Department of Gynecology and Obstetrics, Qilu Hospital of Shandong University, Jinan, Shandong 250012 China; 3 Clinical Epidemiology Unit, Qilu Hospital of Shandong University, Jinan, Shandong 250012 China; 4 Clinical Research Center, Shandong University, Jinan, Shandong 250012 China

**Keywords:** Rheumatoid arthritis, Adverse pregnancy outcomes, Bidirectional two-sample mendelian randomization

## Abstract

**Background:**

There is growing evidence of bidirectional associations between rheumatoid arthritis and adverse pregnancy outcomes (APOs) in observational studies, but little is known about the causal direction of these associations. Therefore, we explored the potential causal relationships between rheumatoid arthritis and APOs using a bidirectional two-sample Mendelian randomization (MR) in European and Asian populations.

**Methods:**

We conducted a bidirectional two-sample Mendelian randomization analysis using available summary statistics from released genome-wide association studies. Summary statistics for instrument–outcome associations were retrieved from two separate databases for rheumatoid arthritis and adverse pregnancy outcomes, respectively. The inverse-variance weighted method was used as the primary MR analysis, and cML-MA-BIC was used as the supplementary analysis. MR-Egger, MR pleiotropy residual sum and outlier (MR-PRESSO), and Cochran Q statistic method were implemented as sensitivity analyses approach to ensure the robustness of the results.

**Results:**

Our study showed that a higher risk of rheumatoid arthritis in the European population was associated with gestational hypertension (OR: 1.04, 95%CI: 1.02–1.06), pre-eclampsia (OR: 1.06, 95%CI: 1.01–1.11), fetal growth restriction (OR: 1.08, 95%CI: 1.04–1.12), preterm delivery (OR:1.04, 95%CI: 1.01–1.07). Furthermore, we found no evidence that APOs had causal effects on rheumatoid arthritis in the reverse MR analysis. No association between rheumatoid arthritis and APOs was found in East Asian population. There was no heterogeneity or horizontal pleiotropy.

**Conclusions:**

This MR analysis provides the positive causal association from rheumatoid arthritis to gestational hypertension, pre-eclampsia, fetal growth restriction and preterm delivery genetically. It highlights the importance of more intensive prenatal care and early intervention among pregnant women with rheumatoid arthritis to prevent potential adverse obstetric outcomes.

**Supplementary Information:**

The online version contains supplementary material available at 10.1186/s12884-024-06698-3.

## Introduction

Rheumatoid arthritis is a common chronic autoimmune disease, which characterized by synovial inflammation, autoantibody production, cartilage and bone destruction, and systemic features, including cardiovascular, pulmonary, psychological, and skeletal disorders [1]. Certain risk factors are known to be associated with an increased likelihood of developing rheumatoid arthritis [2]. First, a positive family history of rheumatoid arthritis increases the risk by approximately three to five times, implicating genetic factors in its pathogenesis [3]. Then, the development of rheumatoid arthritis is associated with environmental factors. Known risk factors include smoking and low socioeconomic status or education. In addition, rheumatoid arthritis can be linked to hormone levels. The global prevalence of rheumatoid arthritis is approximately 0.5–1.0%, and 2 to 3 times more common in women than in men. Several studies have shown that autoimmune diseases are more prevalent in females and are one of the fourth leading causes of disability for women [4, 5]. Rheumatoid arthritis can affect individuals of any age [2] and its incidence of increases with age [6]. Rheumatoid arthritis is a significant global public health challenge. Therefore, early identification and treatment of the disease is vital, especially among females, to minimize the persistent impact of this condition.

Poor maternal and neonatal health remain a recognized public issue and is crucial for sustainable development worldwide [7]. Adverse pregnancy outcomes (APOs) significantly contribute to maternal and neonatal mortality. APOs refer to all pathological complications related to pregnancy and childbirth [8], such as gestational hypertension, pre-eclampsia, preterm delivery, gestational diabetes, fetal growth restriction, delivering a small-for-gestational age baby, placental abruption, and pregnancy loss [9]. The World Health Organization (WHO) reported that in 2020, nearly 800 women died each day due to complications during pregnancy or childbirth [10], and approximately 13.4 million babies were born prematurely [11]. It is clear that preterm birth is currently the primary cause of child deaths [11]. Furthermore, it is estimated that 23 million miscarriages occur worldwide each year, which was equivalent to 44 miscarriages every minute. The pooled risk of miscarriage was 15.3% (95% CI: 12.5–18.7%) of all recognized pregnancies [12]. However, a significant number of adverse maternal and neonatal outcomes can be prevented in advance. Preventing APOs are therefore essential for preserving and extending a healthy lifespan among women.

Previous studies have shown inconsistent results regarding pregnancy-related hypertension and pre-eclampsia/eclampsia in pregnant women with rheumatoid arthritis. Some studies have found a positive association with these complications in pregnant women with rheumatoid arthritis [5, 13, 14], and a retrospective study suggested that women with rheumatoid arthritis had a modestly increased risk for preterm birth and pre-eclampsia [15], while other studies did not find the same associations [16, 17]. Furthermore, there are no articles examining the relationship between rheumatoid arthritis and ectopic pregnancy. Previous studies have shown an association between APOs, such as hyperemesis gravidarum, pre-eclampsia and gestational hypertension, and an increased subsequent risk of developing rheumatoid arthritis [18, 19]. In contrast, an analysis of a prospective case-control study of women who had been pregnant found no statistically significant differences in any APOs, including spontaneous abortion and stillbirth, between rheumatoid arthritis cases and controls [20]. Associations between other APOs and the subsequent risk of rheumatoid arthritis have not been explored. Therefore, a clear assessment of the causality and direction of these associations will help in understanding the disease and contribute to more targeted treatment.

The most important advantage of Mendelian randomization (MR) is that it uses genetic variation as an instrumental variable (IV) to estimate the causal relationship directly and accurately between the exposure phenotype and the outcome phenotype. This approach is independent of external environmental and social behavioral factors, thus overcoming the confounding factors inherent in observational studies, and is a long-term and stable exposure factor [21]. This approach is essentially equivalent to a randomized clinical trial [22].

In this study, we applied a bidirectional two-sample MR analysis to investigate the potential bidirectional causal association between rheumatoid arthritis and APOs, so as to provide evidence for the prevention and control of these diseases.

## Methods

### Study design

**Fig. 1:**
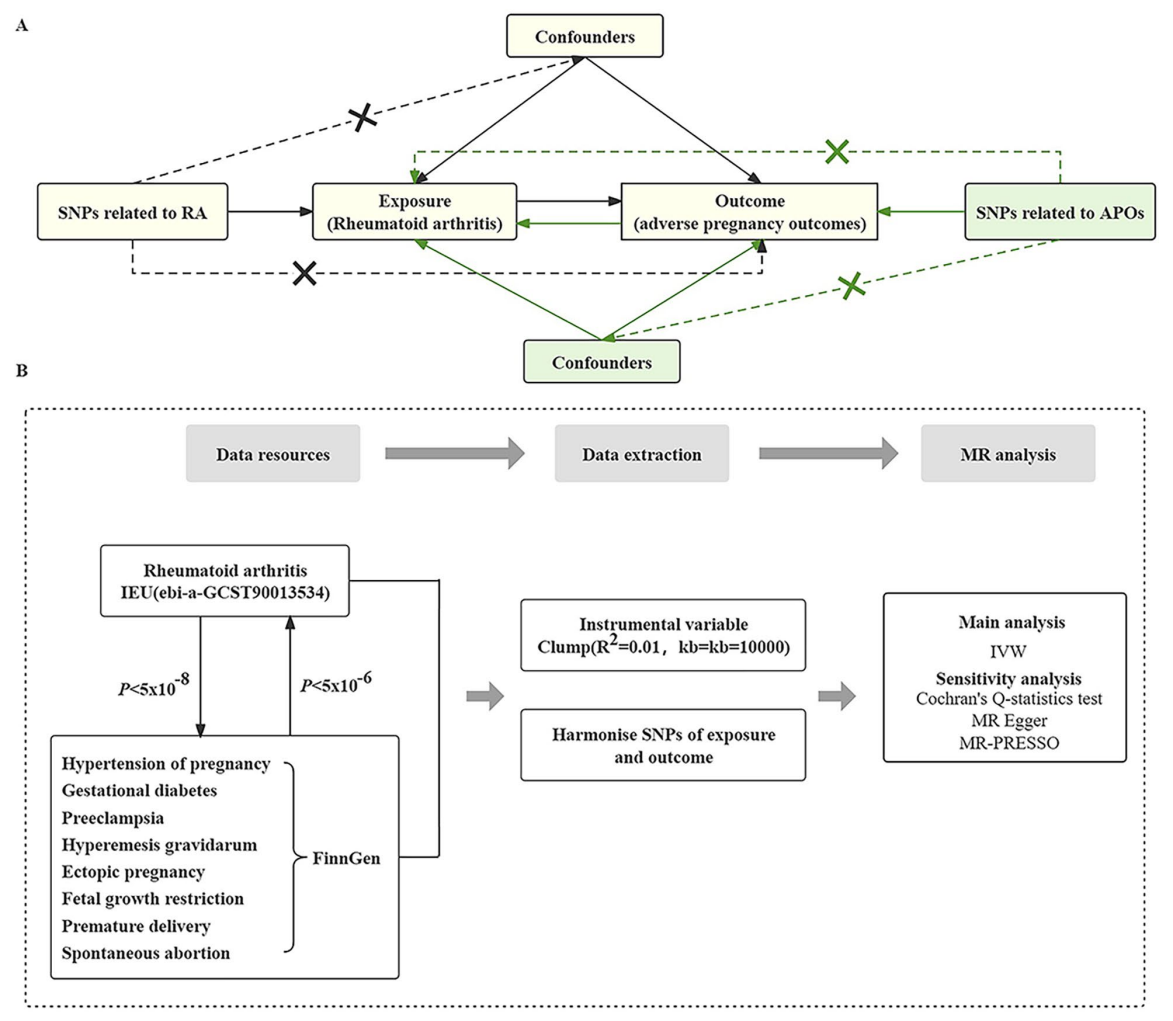
Schematic overview of the study design. **[A]** Mendelian randomization illustration. **[B]** The research design and framework of our study

We performed a bidirectional two-sample MR analysis using data from genome-wide association studies (GWAS) to investigate the relationships between rheumatoid arthritis and APOs, including gestational hypertension, gestational diabetes, pre-eclampsia, hyperemesis gravidarum, ectopic pregnancy, fetal growth restriction, preterm delivery and spontaneous abortion. Single nucleotide polymorphisms (SNPs) linked to exposure were used as genetic instrumental variables (IVs). MR analysis is based on three core assumptions [23]: (a) There is a strong link between IVs and exposure; (b) There are no unmeasured confounders of the associations between genetic IVs and outcome; (c) The genetic IVs influence the outcome only through the exposures and not via other biological pathways. The three prerequisites for IVs in our study were summarized in Fig. 1A. The flowchart illustrating the study design and the process of our MR analysis in this study was shown in Fig. 1B.

### Data sources

We used large-scale publicly available genome-wide association studies (GWAS) summary data to conduct MR analyses of European and East Asian populations. In European populations, GWAS dataset for rheumatoid arthritis was obtained from the IEU Open GWAS project (https://gwas.mrcieu.ac.uk/), with corresponding GWAS IDs of ebi-a-GCST90013534. The GWAS study conducted a large-scale meta-analysis on RA with summary association data from East Asian and European cohorts comprising 22,628 rheumatoid arthritis cases and 288,664 controls (14,361 cases and 43,923 controls in the European population) [24]. Summary-level genetic data for APOs were obtained from the Finnegan study (https://www.finngen.fi/en, R9 released in 2023). FinnGen combines imputed genotype data generated from newly collected and legacy samples from Finnish biobanks and digital health record data from Finnish health registries, with the aim of providing new insights into disease genetics [25]. Among East Asian populations, Summary data on rheumatoid arthritis were obtained from a GWAS study in China [26]. APOs including pre-eclampsia (ID: ebi-a-GCST90018686) [27], ectopic pregnancy (ID: ebi-a-GCST90018617) [27], and spontaneous abortion (ID: ebi-a-GCST90018566) [27] were obtained from the IEU project, using the GWAS study conducted by the BioBank Japan. Table 1 shows the summary statistics of the genetic variants associated with these traits.

**Table 1:** The summary of rheumatoid arthritis and adverse pregnancy outcomes

Trait	Population	Sources	Cases	Controls	No.SNP in MR*
Rheumatoid arthritis	European	IEU (ebi-a-GCST90013534) [24]	14,361	43,923	78
	East Asia	Lei Jiang et al [26]	952	943	5
**Adverse pregnancy outcomes**					
Gestational hypertension	European	FinnGen (O15_HYPTENSPREG)	14,727	196,143	25
Gestational diabetes	European	FinnGen (GEST_DIABETES)	13,039	197,831	52
Pre-eclampsia	European	FinnGen (O15_PREECLAMPS)	6,663	194,266	31
	East Asia	IEU (ebi-a-GCST90018686) [27]	123	81,962	5
Hyperemesis gravidarum	European	FinnGen (O15_EXCESS_VOMIT_PREG)	2,361	179,899	19
Ectopic pregnancy	European	FinnGen (O15_PREG_ECTOP)	5,648	149,622	9
	East Asia	IEU (ebi-a-GCST90018617) [27]	605	82,156	4
Fetal growth restriction	European	FinnGen (O15_POOR_FETGRO)	3,558	207,312	10
Preterm delivery	European	FinnGen (O15_PRETERM)	8,507	162,777	8
Spontaneous abortion	European	FinnGen (O15_ABORT_SPONTAN)	16,906	149,622	11
	East Asia	IEU (ebi-a-GCST90018566) [27]	136	82,625	0

SNPs, single nucleotide polymorphisms; MR: Mendelian randomization

* When rheumatoid arthritis was used as an exposure, the significance threshold for SNPs was set at *P* < 5 × 10^− 8^. And when adverse pregnancy outcomes were used as an exposure, the significance threshold for SNPs was set at *P* < 5 × 10^− 6.^

### Instruments selection

SNPs were used as instrumental variables (IVs). When rheumatoid arthritis was used as exposure, the significance threshold for SNPs was set at *P* < 5 × 10^− 8^. However, when APOs were used as exposures, the significance threshold was expanded to *P* < 5 × 10^− 6^. This adjustment was made to ensure that an adequate number of SNPs were available for the heterogeneity test and pleiotropy test. Secondly, to exclude SNPs that were in strong linkage disequilibrium (LD), we performed the clumping procedure with R^2^ < 0.01 and clump distance = 10,000 kb. The strength of IVs was assessed by calculating the F-statistic using the formula F = R^2^(N-2)/(1-R^2^), where R^2^ represents the proportion of variance in the phenotype explained by the genetic variants, and N is the sample size of the GWAS for the exposure [28]. The R^2^ of instrumental variables can be calculated using the formula below [29]: R^2^ = 2 × (1-MAF)×MAF × β^2^. whereas for the extended 10 SNP instruments, we used: R^2^=[2×EAF×(1-EAF)×β^2^]/[2×EAF×(1-EAF)×β^2^ + 2×EAF×(1-EAF)×N×SE^2^], where β indicates the estimated genetic effect of the SNP on exposure, EAF denotes the effect allele frequency, MAF represents the other effect allele frequency, Ν is the sample size of the GWAS, and SE is the standard error of the genetic effect. The data mentioned above can be obtained from the original summary data. We retained the SNPs with F > 10 as the final genetic variables to avoid the risk of selecting weak instrumental variables.

### Statistical analysis

To investigate the causal relationship between exposure and outcome, bidirectional two-sample MR analyses were performed using several methods, including inverse variance weighting (IVW), weighted median, MR-Egger regression, simple mode, and weighted modal methods. We used the fixed-effects inverse-variance weighting (IVW) as the primary analytical method. However, when heterogeneity is observed, the random-effects IVW was used. In addition, Mendelian randomization based on constrained maximum likelihood and model averaging Bayesian information criterion (cML-MA-BIC) was employed to control correlated and uncorrelated pleiotropic effects. Because this method combines the advantages of maximum likelihood estimation and model averaging, it allows better control the Type-I error rate in the estimation [30]. The heterogeneity was quantified using the *P*-value of Cochran’s Q-statistics test, which quantifies the extent to which any differences in the individual effect sizes among the selected genetic variants are due to actual differences between SNPs rather than sampling error. A *P*-value of less than 0.05 implies the presence of heterogeneity.

In addition, we conducted a sensitivity analysis to evaluate the robustness of the association. To assess the influence of horizontal pleiotropy, the *P*-value of the MR-Egger regression intercept was used to identify and adjust for bias resulting from directional pleiotropy. When *P* < 0.05, it indicated that there was significant pleiotropy bias. Then, the MR pleiotropy residual sum and outlier (MR-PRESSO) test was conducted to detect and rectify any horizontal pleiotropic outliers. This was done to obtain accurate results by removing any outliers.

The associations between rheumatoid arthritis and APOs were presented using ORs along with their 95% confidence intervals (CIs). We adjusted for multiple testing using a Bonferroni-corrected threshold of *P* < 0.0063 (*P* < 0.05/8). The *P*-values ranging from 0.0063 to 0.05 were considered to indicate suggestive associations. The study was adhered to the STROBE-MR guidelines [31]. All analyses were performed using R statistical software (Version 4.2.3; https://www.r-project.org/). MR analyses were performed using the R-based “TwoSampleMR”,“MRPROSSO” and “MRcML” packages, and forest plots were generated using the “forestplot” package.

## Results

### Causal effects of rheumatoid arthritis on APOs risk in the European population

For IVs used for rheumatoid arthritis, all the F-statistics were greater than 10, indicating no presence of weak instrumental bias. After removing linkage disequilibrium (LD) and anomalous outliers, we incorporated 78 SNPs with a significant *P*-value less than 5 × 10^− 8^ as IVs for rheumatoid arthritis, Detailed information about exposure was listed in the Table S1.

**Fig. 2:**
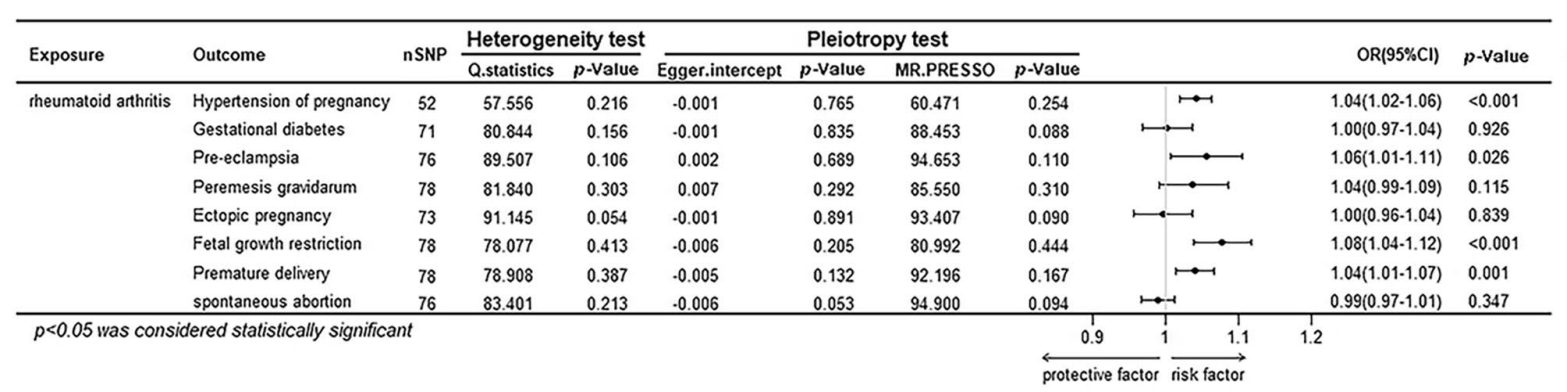
Total causal effects of rheumatoid arthritis on APOs.

Figure 2 illustrated the primary results of the causal relationship between rheumatoid arthritis and APOs. Among the main results of using IVW, we observed a causal association between rheumatoid arthritis and gestational hypertension, pre-eclampsia, fetal growth restriction and preterm delivery, with corresponding odds ratio (OR) =1.04 (95%CI: 1.02–1.06; *P* = 9.89 × 10^− 5^), 1.06 (95%CI: 1.01–1.11; *P* = 6.47 × 10^− 5^), 1.08 (95%CI: 1.04–1.12; *P* = 7.20 × 10^− 5^), 1.04 (95%CI: 1.01–1.07; *P* = 0.001). respectively, none of them changed their significance levels after applying the Bonferroni correction. Detailed results of the MR analyses using different methods, such as MR Egger, weighted median, simple mode, weighted mode, were presented in the Table S2. The methods mentioned above are consistent with IVW, except for the Simple model. In addition, the complementary method cML-MA-BIC also confirmed the causal association of the above rheumatoid arthritis with hypertension in pregnancy, pre-eclampsia, and foetal growth restriction (Table S3).

In the sensitivity analyses (Table S4), heterogeneity was evaluated using Cochran’s Q test, which was not found to be significant (*P* > 0.05). The MR Egger intercept test did not observe a significant pleiotropy, with *P*-values ranging from 0.053 to 0.891. And no outlier SNPs were identified by using MR-PRESSO in our study, with *P*-values ranging from 0.088 to 0.444.

### Causal effects of APOs on rheumatoid arthritis risk in the European population

Based on the same screening criteria, 25 SNPs were selected as IVs for gestational hypertension, 52 SNPs for gestational diabetes, 31 SNPs for pre-eclampsia, 19 SNPs for hyperemesis gravidarum, 9 SNPs for ectopic pregnancy, 10 SNPs for fetal growth restriction, 8 SNPs for preterm delivery, and 11 SNPs for spontaneous abortion. A summary and detailed information about the SNPs for each exposure were presented in the Table S5.

**Fig. 3:**
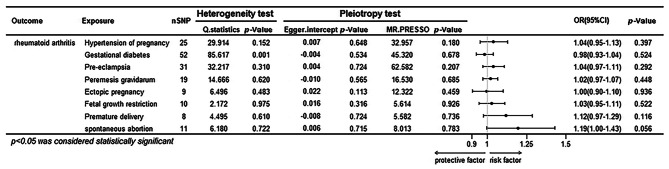
Total causal effects of APOs on rheumatoid arthritis

In general, the primary analysis using IVW did not reveal a statistically significant relationship between an increase in the risk of having APOs and an increased risk of rheumatoid arthritis (Table S6, Fig. 3). While the other five MR methods (weighted median, MR-Egger regression, simple mode, weighted modal methods and cML-MA-BIC) affirmed the identical causal effect of APOs on rheumatoid arthritis (Table S7). Neither the MR-Egger intercept test nor Cochran’s Q statistic revealed any evidence of directional pleiotropy or heterogeneity. In addition, the MR-PRESSO global test did not reveal any evidence of horizontal pleiotropy (all *P* > 0.18). The results of the sensitivity analyses were shown in Table S8.

### Rheumatoid arthritis and APOs risk in the east Asian population

There were a total of 5 SNPs with *P* < 5 × 10^− 6^, and a total of 3 SNPs were included after removing the LD (rs12525220, rs7748270). A summary and detailed information about the SNPs were presented in the Table S9. The results failed to reveal any discernible causal relationship between RA and APOs, as confirmed by the statistical insignificance shown in Table S10 (*P* > 0.0063). cML-MA-BIC Similarly did not find any significant difference. Cochran’s Q test and MR-Egger (Table S11) showed no significant heterogeneity or horizontal pleiotropy.

In the reverse Mendelian randomization analysis, we excluded the effect of LD and merged the results with APOs. However, we did not find any SNPs that were closely associated with rheumatoid arthritis. Due to the lack of the related SNPs we were unable to conduct analysis.

## Discussion

### Main findings

In this study, we used a bidirectional two-sample Mendelian randomization analysis to investigate the causal relationship between rheumatoid arthritis and adverse pregnancy outcomes in European and East Asian populations. This study indicated that in European population, rheumatoid arthritis was associated with the increased risk of gestational hypertension, pre-eclampsia, fetal growth restriction and preterm delivery. And these associations remained consistent even after multiple corrections were applied to the data. Furthermore, our study did not find evidence of causal associations of APOs on the increased risk of rheumatoid arthritis. It was also evident from the sensitivity analysis that the results of this study were robust and reliable. In the East Asian population, we did not find any association between rheumatoid arthritis and APOs.

### Interpretation

Our findings in bidirectional Mendelian randomization of the association of RA with gestational hypertension and preeclampsia are consistent with previous observational studies [5, 32]. A systematic review [33] also found that women with rheumatoid arthritis tended to have a higher risk of maternal and neonatal complications compared to the general pregnant population. There have been observational studies [34, 35] and a meta-analysis [36] demonstrated that maternal rheumatoid arthritis during pregnancy was associated with a significantly increased risk of preterm birth and low birth weight in the fetus. The aforementioned studies provide support for our findings regarding the causal associations between genetically predicted rheumatoid arthritis and gestational hypertension, pre-eclampsia, fetal growth restriction, and preterm delivery. There was certainly a good deal of evidence that rheumatoid arthritis was the risk factor for small for gestational age infants [5, 13, 37]. However, due to the limited number of GWAS studies, we have been unable to find a suitable database.

Several possible mechanisms have been proposed to explain the association between rheumatoid arthritis and gestational hypertension, pre-eclampsia, fetal growth restriction and preterm delivery. First, in patients with rheumatoid arthritis, the CD4 protein on helper T lymphocytes is activated, which then stimulates monocytes, macrophages, and fibroblast-like synoviocytes. This activation can result in an increased release of proinflammatory cytokines, such as interleukin-1 (IL-1), interleukin-6 (IL-6), and tumor necrosis factor-alpha (TNF-α). Additionally, it can lead to a decrease in the release of regulatory and anti-inflammatory cytokines [38]. High levels of proinflammatory cytokines, as well as chronic stress states, can reduce the activity of 11β-HSD2 and result in elevated maternal cortisol levels, which may have potentially deleterious effects on the placenta, leading to preterm birth, low birth weight, and small for gestational age [39, 40]. Secondly, endothelial dysfunction is a common complication of active rheumatoid arthritis. Vasculopathy resulting from endothelial dysfunction may contribute to placental maldevelopment. Maldevelopment of the placenta is associated with unfavorable pregnancy outcomes, such as lower birth weight and hypertension [41]. Endothelial dysfunction is considered the initial stage of atherosclerosis [42], and proinflammatory cytokines implicated in rheumatoid arthritis also contribute to the development of atherosclerosis [43]. Furthermore, in patients with rheumatoid arthritis, the upregulated expression of vascular endothelial growth factor (VEGF), a crucial regulator of endothelial dysfunction [44], may lead to the development of pre-eclampsia during pregnancy [45].

Epidemiologic data suggests that pregnancy-related hormones may influence the link between reproduction and the risk of rheumatoid arthritis. Excessive level of female hormones, such as estrogen and progesterone, may be protective against the development of rheumatoid arthritis [46]. During pregnancy, when estradiol and progesterone levels are high, women have a reduced risk of developing rheumatoid arthritis [47]. Hormone levels return to a non-pregnant state rapidly after delivery [48], especially during the first 3 months, which appears to be a period of increased risk. A nationwide cohort study in Denmark found that women with hyperemesis gravidarum, gestational hypertension, or pre-eclampsia have a significantly higher risk of developing rheumatoid arthritis [18]. However, a population-based prospective study found that preterm delivery and small-for-gestational-age infants did not appear to have a significant association with subsequent rheumatoid arthritis [49]. Our study also did not find the causal relationship from the gene perspective. This may be due to the lack of individual-level information in the pooled data used, and the fact that the data on rheumatoid arthritis did not include data on its typing, due to which limitation it is difficult to determine a causal association of APOs on rheumatoid arthritis progression.

### Strengths and limitations

This bidirectional two-sample MR study, which investigated the causal association between rheumatoid arthritis and APOs risk, had several strengths. First, the bidirectional two-sample Mendelian randomization analysis can reduce the effects of unknown confounders and small-sample selection bias. The bidirectional analysis ensures the inference of causality between rheumatoid arthritis and APOs in both directions. Furthermore, the study used a large sample size and SNPs from GWAS in European population, which provided sufficient statistical validity to estimate causality. Finally, we applied a series of sensitivity analyses to ensure the consistency and the robustness of causal estimates.

Inevitably, there were several limitations in our study. First, considering the problem of sample overlap, this study did not find a large sample of rheumatoid arthritis -related GWAS studies in East Asian populations, and APOs-related GWAS studies were limited, making it impossible to draw the same clear conclusions as European populations. Secondly, when examining the risk of rheumatoid arthritis associated with APOs, we set the *P*-value threshold for SNPs at *P* < 5 × 10^− 6^ to ensure an adequate number of instrumental variables for heterogeneity and horizontal multivariate tests. This threshold may explain only a small portion of the variability in exposures and could affect the statistical efficacy of the causal estimates. Finally, we were unable to perform subgroup analyses due to the lack of specific information describing the severity of the disease and specific information at the individual level.

## Conclusions

In conclusion, this study confirmed the causal relationship between rheumatoid arthritis and adverse pregnancy outcomes in the European population, that is, rheumatoid arthritis is associated with an increased risk of gestational hypertension, pre-eclampsia, fetal growth restriction, and preterm delivery. It highlights the importance of more intensive prenatal care and early intervention among pregnant women with rheumatoid arthritis to prevent potential adverse obstetric outcomes. However, due to the limitations of the study, further research is warranted.

### Electronic supplementary material

Below is the link to the electronic supplementary material.

Supplementary Material 1

Supplementary Material 2

## Data Availability

Data is provided within the manuscript or supplementary information files. This study uses publicly available datasets, which can be found in the IEU Open Project (https://gwas.mrcieu.ac.uk/), and the FinnGen study (https://www.finngen.fi/en).

## Abbreviations

APOs: Adverse Pregnancy Outcomes
LD: Linkage Disequilibrium
MR: Mendelian randomization
SNP: Single-nucleotide polymorphism
GWAS: Genome-Wide Association Studies
IV: Instrumental Variable
IVW: Inverse Variance-Weighted
MR-PRESSO: MR pleiotropy residual sum and outlier
