# Supplementary material for: Rheumatoid arthritis and adverse pregnancy outcomes: a bidirectional two-sample mendelian randomization study
Source: BMC Pregnancy Childbirth. 2024 Jul 31;24:517. doi: 10.1186/s12884-024-06698-3 (PMC11293129; doi:10.1186/s12884-024-06698-3)
Supplement: Supplementary file 1 — Supplementary Material 1 [file 12884_2024_6698_MOESM1_ESM.docx]

STROBE-MR checklist of recommended items to address in reports of Mendelian randomization studies^[1, 2]^

| Item No. | Section | Checklist item | Relevant text from manuscript |
| --- | --- | --- | --- |
| 1 | **TITLE and ABSTRACT** | Indicate Mendelian randomization (MR) as the study’s design in the title and/or the abstract if that is a main purpose of the study | Rheumatoid Arthritis and Adverse Pregnancy Outcomes: A Bidirectional Two-Sample Mendelian Randomization Study |
|  |  |  |  |
| 2 | **Background** | Explain the scientific background and rationale for the reported study. What is the exposure? Is a potential causal relationship between exposure and outcome plausible? Justify why MR is a helpful method to address the study question | Previous studies have shown inconsistent results regarding pregnancy-related hypertension and pre-eclampsia/eclampsia in pregnant women with rheumatoid arthritis. Some studies have found a positive association with these complications in pregnant women with rheumatoid arthritis, and a retrospective study suggested that women with rheumatoid arthritis had a modestly increased risk for preterm birth and pre-eclampsia while other studies did not find the same associations. Furthermore, there are no articles examining the relationship between rheumatoid arthritis and ectopic pregnancy. Previous studies have shown an association between APOs, such as hyperemesis gravidarum, pre-eclampsia and gestational hypertension, and an increased subsequent risk of developing rheumatoid arthritis. In contrast, an analysis of a prospective case-control study of women who had been pregnant found no statistically significant differences in any APOs, including spontaneous abortion and stillbirth, between rheumatoid arthritis cases and controls. Associations between other APOs and the subsequent risk of rheumatoid arthritis have not been explored. Therefore, a clear assessment of the causality and direction of these associations will help in understanding the disease and contribute to more targeted treatment. |
| 3 | **Objectives** | State specific objectives clearly, including pre-specified causal hypotheses (if any). State that MR is a method that, under specific assumptions, intends to estimate causal effects | In this study, we applied a bidirectional two-sample MR analysis to investigate the potential bidirectional causal association between rheumatoid arthritis and APOs, so as to provide evidence for the prevention and control of these diseases. |
|  |  |  |  |
| 4 | **Study design and data sources** | Present key elements of the study design early in the article. Consider including a table listing sources of data for all phases of the study. For each data source contributing to the analysis, describe the following: |  |
|  |  | a) Setting: Describe the study design and the underlying population, if possible. Describe the setting, locations, and relevant dates, including periods of recruitment, exposure, follow-up, and data collection, when available | We used large-scale publicly available genome-wide association studies (GWAS) summary data to conduct MR analyses of European and East Asian populations. In European populations, GWAS dataset for rheumatoid arthritis was obtained from the IEU Open GWAS project (https://gwas.mrcieu.ac.uk/), with corresponding GWAS IDs of ebi-a-GCST90013534. The GWAS study conducted a large-scale meta-analysis on RA with summary association data from East Asian and European cohorts comprising 22,628 rheumatoid arthritis cases and 288,664 controls (14,361 cases and 43,923 controls in the European population)(24). Summary-level genetic data for APOs were obtained from the Finnegan study (https://www.finngen.fi/en, R9 released in 2023). FinnGen combines imputed genotype data generated from newly collected and legacy samples from Finnish biobanks and digital health record data from Finnish health registries, with the aim of providing new insights into disease genetics (25). Among East Asian populations, Summary data on rheumatoid arthritis were obtained from a GWAS study in China(26). APOs including pre-eclampsia (ID: ebi-a-GCST90018686)(27), ectopic pregnancy (ID: ebi-a-GCST90018617)(27), and spontaneous abortion (ID:ebi-a-GCST90018566)(27) were obtained from the IEU project, using the GWAS study conducted by the BioBank Japan. |
|  |  | b) Participants: Give the eligibility criteria, and the sources and methods of selection of participants. Report the sample size, and whether any power or sample size calculations were carried out prior to the main analysis | In European populations, GWAS dataset for rheumatoid arthritis was obtained from the IEU Open GWAS project (https://gwas.mrcieu.ac.uk/), with corresponding GWAS IDs of ebi-a-GCST90013534. Eunji Ha et al. published the results of a GWAS meta-analysis of RA related loci in this database in 2020(24). |
|  |  | c) Describe measurement, quality control and selection of genetic variants | SNPs were used as instrumental variables (IVs). When rheumatoid arthritis was used as exposure, the significance threshold for SNPs was set at P<5×10-8. However, when APOs were used as exposures, the significance threshold was expanded to P<5×10-6. This adjustment was made to ensure that an adequate number of SNPs were available for the heterogeneity test and pleiotropy test. Secondly, to exclude SNPs that were in strong linkage disequilibrium (LD), we performed the clumping procedure with R2<0.01 and clump distance=10,000kb.  The data mentioned above can be obtained from the original summary data. We retained the SNPs with F>10 as the final genetic variables to avoid the risk of selecting weak instrumental variables. |
|  |  | d) For each exposure, outcome, and other relevant variables, describe methods of assessment and diagnostic criteria for diseases | The GWAS study conducted a large-scale meta-analysis on RA with summary association data from East Asian and European cohorts comprising 22,628 rheumatoid arthritis cases and 288,664 controls (14,361 cases and 43,923 controls in the European population) |
|  |  | e) Provide details of ethics committee approval and participant informed consent, if relevant | Ethical approval was not required because the analyses involved public abstract-level datasets. |
| 5 | **Assumptions** | Explicitly state the three core IV assumptions for the main analysis (relevance, independence and exclusion restriction) as well assumptions for any additional or sensitivity analysis | MR analysis is based on three core assumptions (23): (a) There is a strong link between IVs and exposure; (b) There are no unmeasured confounders of the associations between genetic IVs and outcome; (c) The genetic IVs influence the outcome only through the exposures and not via other biological pathways. The three prerequisites for IVs in our study were summarized in Figure 1A. The flowchart illustrating the study design and the process of our MR analysis in this study was shown in Figure 1B. |
| 6 | **Statistical methods: main analysis** | Describe statistical methods and statistics used |  |
|  |  | a) Describe how quantitative variables were handled in the analyses (i.e., scale, units, model) | This research does not involve any transformations of  quantitative variables. |
|  |  | b) Describe how genetic variants were handled in the analyses and, if applicable, how their weights were selected | To investigate the causal relationship between exposure and outcome, bidirectional two-sample MR analyses were performed using several methods, including inverse variance weighting (IVW), weighted median, MR-Egger regression, simple mode, and weighted modal methods. We used the fixed-effects inverse-variance weighting (IVW) as the primary analytical method. However, when heterogeneity is observed, the random-effects IVW was used. In addition, Mendelian randomization based on constrained maximum likelihood and model averaging Bayesian information criterion (cML-MA-BIC) was employed to control correlated and uncorrelated pleiotropic effects. Because this method combines the advantages of maximum likelihood estimation and model averaging, it allows better control the Type-I error rate in the estimation(30). The heterogeneity was quantified using the P-value of Cochran's Q-statistics test, which quantifies the extent to which any differences in the individual effect sizes among the selected genetic variants are due to actual differences between SNPs rather than sampling error. A P-value of less than 0.05 implies the presence of heterogeneity. |
|  |  | c) Describe the MR estimator (e.g. two-stage least squares, Wald ratio) and related statistics. Detail the included covariates and, in case of two-sample MR, whether  the same covariate set was used for adjustment in the two samples | The associations between rheumatoid arthritis and APOs were presented using ORs along with their 95% confidence intervals (CIs). We adjusted for multiple testing using a Bonferroni-corrected threshold of P<0.0063 (P<0.05/8). The P-values ranging from 0.0063 to 0.05 were considered to indicate suggestive associations. |
|  |  | d) Explain how missing data were addressed | this MR analysis, the issue of missing data was not involved. |
|  |  | e) If applicable, indicate how multiple testing was addressed | We adjusted for multiple testing using a Bonferroni-corrected threshold of P<0.0063 (P<0.05/8). The P-values ranging from 0.0063 to 0.05 were considered to indicate suggestive associations. |
| 7 | **Assessment of assumptions** | Describe any methods or prior knowledge used to assess the assumptions or justify their validity | We retained the SNPs with F>10 as the final genetic variables to avoid the risk of selecting weak instrumental variables.  In addition, Mendelian randomization based on constrained maximum likelihood and model averaging Bayesian information criterion (cML-MA-BIC) was employed to control correlated and uncorrelated pleiotropic effects. Because this method combines the advantages of maximum likelihood estimation and model averaging, it allows better control the Type-I error rate in the estimation (30). |
| 8 | **Sensitivity analyses and additional**  **Analyses** | Describe any sensitivity analyses or additional analyses performed (e.g. comparison of effect estimates from different approaches, independent replication, bias analytic techniques, validation of instruments, simulations) | In addition, we conducted a sensitivity analysis to evaluate the robustness of the association. To assess the influence of horizontal pleiotropy, the P-value of the MR-Egger regression intercept was used to identify and adjust for bias resulting from directional pleiotropy. When P<0.05, it indicated that there was significant pleiotropy bias. Then, the MR pleiotropy residual sum and outlier (MR-PRESSO) test was conducted to detect and rectify any horizontal pleiotropic outliers. This was done to obtain accurate results by removing any outliers. |
| 9 | **Software and preregistration** |  |  |
|  |  | a) Name statistical software and package(s), including version and settings used | All analyses were performed using R statistical software (Version 4.2.3; https://www.r-project.org/). MR analyses were performed using the R-based "TwoSampleMR","MRPROSSO" and “MRcML” packages, and forest plots were generated using the "forestplot" package. |
|  |  | b) State whether the study protocol and details were pre-registered (as well as when and where) | This study was not pre-registered with the study protocol and details. |
|  | **RESULTS** |  |  |
| 10 | **Descriptive data** |  | Details see Table1 |
|  |  | a) Report the numbers of individuals at each stage of included studies and reasons for exclusion. Consider use of a flow diagram | Summary data on exposure and outcomes are shown Supplementary Tables (1, 5, 9). |
|  |  | b) Report summary statistics for phenotypic exposure(s), outcome(s), and other relevant variables (e.g. means, SDs, proportions) | For IVs used for rheumatoid arthritis, all the F-statistics were greater than 10, indicating no presence of weak instrumental bias. After removing linkage disequilibrium (LD) and anomalous outliers, we incorporated 78 SNPs with a significant P-value less than 5×10-8 as IVs for rheumatoid arthritis, Detailed information about exposure was listed in the Table S1.  Based on the same screening criteria, 25 SNPs were selected as IVs for gestational hypertension, 52 SNPs for gestational diabetes, 31 SNPs for pre-eclampsia, 19 SNPs for hyperemesis gravidarum, 9 SNPs for ectopic pregnancy, 10 SNPs for fetal growth restriction, 8 SNPs for preterm delivery, and 11 SNPs for spontaneous abortion. A summary and detailed information about the SNPs for each exposure were presented in the Table S5. |
|  |  | c) If the data sources include meta-analyses of previous studies, provide the assessments of heterogeneity across these studies | We retained the SNPs with F>10 as the final genetic variables to avoid the risk of selecting weak instrumental variables. |
|  |  | d) For two-sample MR:  i. Provide justification of the similarity of the genetic variant-exposure associations between the exposure and outcome samples  ii. Provide information on the number of individuals who overlap between the exposure and outcome studies | detailed data on the number of individuals in the exposure and outcome samples are provided in Supplementary Tables (1, 5, 9). |
| 11 | **Main results** |  |  |
|  |  | a) Report the associations between genetic variant and exposure, and between genetic variant and outcome, preferably on an interpretable scale | Among the main results of using IVW, we observed a causal association between rheumatoid arthritis and gestational hypertension, pre-eclampsia, fetal growth restriction and preterm delivery, with corresponding odds ratio (OR) =1.04 (95%CI: 1.02-1.06; P=9.89×10-5), 1.06 (95%CI: 1.01-1.11; P=6.47×10-5), 1.08 (95%CI: 1.04-1.12; P=7.20×10-5), 1.04 (95%CI: 1.01-1.07; P=0.001).  In general, the primary analysis using IVW did not reveal a statistically significant relationship between an increase in the risk of having APOs and an increased risk of rheumatoid arthritis (Table S6, Figure 3).  The results failed to reveal any discernible causal relationship between RA and APOs, as confirmed by the statistical insignificance shown in Table S10 (p> 0.0063). |
|  |  | b) Report MR estimates of the relationship between exposure and outcome, and the measures of uncertainty from the MR analysis, on an interpretable scale, such as odds ratio or relative risk per SD difference | Mengelian randomization estimation reports are detailed in Supplementary Tables (2,3,6,7,10). |
|  |  | c) If relevant, consider translating estimates of relative risk into absolute risk for a meaningful time period | The calculation of absolute risk is detailed in Supplementary Tables (2,3,6,7,10). |
|  |  | d) Consider plots to visualize results (e.g. forest plot, scatterplot of associations between genetic variants and outcome versus between genetic variants and exposure) | See Figure 2,3 |
| 12 | **Assessment of**  **assumptions** |  |  |
|  |  | a) Report the assessment of the validity of the assumptions |  |
|  |  | b) Report any additional statistics (e.g., assessments of heterogeneity across genetic variants, such as *I^2^*, Q statistic or E-value) | See Supplementary Table 4,8,11 |
| 13 | **Sensitivity**  **analyses and**  **additional**  **analyses** |  |  |
|  |  | a) Report any sensitivity analyses to assess the robustness of the main results to violations of the assumptions | In the sensitivity analyses (Table S4), heterogeneity was evaluated using Cochran's Q test, which was not found to be significant (P>0.5). The MR Egger intercept test did not observe a significant pleiotropy, with P-values ranging from 0.053 to 0.891. And no outlier SNPs were identified by using MR-PRESSO in our study, with P-values ranging from 0.088 to 0.444.  Neither the MR-Egger intercept test nor Cochran's Q statistic revealed any evidence of directional pleiotropy or heterogeneity. In addition, the MR-PRESSO global test did not reveal any evidence of horizontal pleiotropy (all P>0.18). |
|  |  | b) Report results from other sensitivity analyses or additional analyses | The methods mentioned above are consistent with IVW, except for the Simple model. In addition, the complementary method cML-MA-BIC also confirmed the causal association of the above rheumatoid arthritis with hypertension in pregnancy, pre-eclampsia, and foetal growth restriction (Table S3).  While the other five MR methods (weighted median, MR-Egger regression, simple mode, weighted modal methods and cML-MA-BIC) affirmed the identical causal effect of APOs on rheumatoid arthritis (Table S7).  cML-MA-BIC Similarly did not find any significant difference. |
|  |  | c) Report any assessment of direction of causal relationship (e.g., bidirectional MR) | In the reverse Mendelian randomization analysis, we excluded the effect of LD and merged the results with APOs. However, we did not find any SNPs that were closely associated with rheumatoid arthritis. Due to the lack of the related SNPs we were unable to conduct analysis. |
|  |  | d) When relevant, report and compare with estimates from non-MR analyses | This study does not involve non-MR studies. |
|  |  | e) Consider additional plots to visualize results (e.g., leave-one-out analyses) | No |
|  | **DISCUSSION** |  |  |
| 14 | **Key results** | Summarize key results with reference to study objectives | In this study, we used a bidirectional two-sample Mendelian randomization analysis to investigated the causal relationship between rheumatoid arthritis and adverse pregnancy outcomes in European and East Asian populations. using a bidirectional two-sample Mendelian randomization analysis. This study indicated that in European population, genetically predicted rheumatoid arthritis was associated with the increased risk of gestational hypertension, pre-eclampsia, fetal growth restriction and preterm delivery. And these associations remained consistent even after multiple corrections were applied to the data. Furthermore, our study did not find evidence of causal associations of genetically predicted APOs on the increased risk of rheumatoid arthritis. It was also evident from the sensitivity analysis that the results of this study were robust and reliable. In the East Asian population, we did not find any association between rheumatoid arthritis and APOs. However, due to the small sample size of the GWAS study used, the results used as exploratory results. |
| 15 | **Limitations** | Discuss limitations of the study, taking into account the validity of the IV assumptions, other sources of potential bias, and imprecision. Discuss both direction and magnitude of any potential bias and any efforts to address them | Inevitably, there were several limitations in our study. First, considering the problem of sample overlap, this study did not find a large sample of rheumatoid arthritis -related GWAS studies in East Asian populations, and APOs-related GWAS studies were limited, making it impossible to draw the same clear conclusions as European populations. Secondly, when examining the risk of rheumatoid arthritis associated with APOs, we set the P-value threshold for SNPs at P<5×10-6 to ensure an adequate number of instrumental variables for heterogeneity and horizontal multivariate tests. This threshold may explain only a small portion of the variability in exposures and could affect the statistical efficacy of the causal estimates. Finally, we were unable to perform subgroup analyses due to the lack of specific information describing the severity of the disease and specific information at the individual level. |
| 16 | **Interpretation** |  |  |
|  |  | a) Meaning: Give a cautious overall interpretation of results in the context of their limitations and in comparison with other studies | Our findings in bidirectional Mendelian randomization of the association of RA with gestational hypertension and preeclampsia are consistent with previous observational studies (5, 32). A systematic review (33) also found that women with rheumatoid arthritis tended to have a higher risk of maternal and neonatal complications compared to the general pregnant population. There have been observational studies (34, 35) and a meta-analysis (36) demonstrated that maternal rheumatoid arthritis during pregnancy was associated with a significantly increased risk of preterm birth and low birth weight in the fetus. The aforementioned studies provide support for our findings regarding the causal associations between genetically predicted rheumatoid arthritis and gestational hypertension, pre-eclampsia, fetal growth restriction, and preterm delivery. There was certainly a good deal of evidence that rheumatoid arthritis was the risk factor for small for gestational age infants (5, 13, 37). However, due to the limited number of GWAS studies, we have been unable to find a suitable database. |
|  |  | b) Mechanism: Discuss underlying biological mechanisms that could drive a potential causal relationship between the investigated exposure and the outcome, and whether the gene-environment equivalence assumption is reasonable. Use causal language carefully, clarifying that IV estimates may provide causal effects only under certain assumptions | Several possible mechanisms have been proposed to explain the association between rheumatoid arthritis and gestational hypertension, pre-eclampsia, fetal growth restriction and preterm delivery. First, in patients with rheumatoid arthritis, the CD4 protein on helper T lymphocytes is activated, which then stimulates monocytes, macrophages, and fibroblast-like synoviocytes. This activation can result in an increased release of proinflammatory cytokines, such as interleukin-1 (IL-1), interleukin-6 (IL-6), and tumor necrosis factor-alpha (TNF-α). Additionally, it can lead to a decrease in the release of regulatory and anti-inflammatory cytokines (38). High levels of proinflammatory cytokines, as well as chronic stress states, can reduce the activity of 11β-HSD2 and result in elevated maternal cortisol levels, which may have potentially deleterious effects on the placenta, leading to preterm birth, low birth weight, and small for gestational age (39, 40). Secondly, endothelial dysfunction is a common complication of active rheumatoid arthritis. Vasculopathy resulting from endothelial dysfunction may contribute to placental maldevelopment. Maldevelopment of the placenta is associated with unfavorable pregnancy outcomes, such as lower birth weight and hypertension (41). Endothelial dysfunction is considered the initial stage of atherosclerosis (42), and proinflammatory cytokines implicated in rheumatoid arthritis also contribute to the development of atherosclerosis (43). Furthermore, in patients with rheumatoid arthritis, the upregulated expression of vascular endothelial growth factor (VEGF), a crucial regulator of endothelial dysfunction (44), may lead to the development of pre-eclampsia during pregnancy (45). |
|  |  | c) Clinical relevance: Discuss whether the results have clinical or public policy relevance, and to what extent they inform effect sizes of possible interventions | Epidemiologic data suggests that pregnancy-related hormones may influence the link between reproduction and the risk of rheumatoid arthritis. Excessive level of female hormones, such as estrogen and progesterone, may be protective against the development of rheumatoid arthritis (46). During pregnancy, when estradiol and progesterone levels are high, women have a reduced risk of developing rheumatoid arthritis (47). Hormone levels return to a non-pregnant state rapidly after delivery (48), especially during the first 3 months, which appears to be a period of increased risk. A nationwide cohort study in Denmark found that women with hyperemesis gravidarum, gestational hypertension, or pre-eclampsia have a significantly higher risk of developing rheumatoid arthritis (18). However, a population-based prospective study found that preterm delivery and small-for-gestational-age infants did not appear to have a significant association with subsequent rheumatoid arthritis (49). Our study also did not find the causal relationship from the gene perspective. This may be due to the lack of individual-level information in the pooled data used, and the fact that the data on rheumatoid arthritis did not include data on its typing, due to which limitation it is difficult to determine a causal association of APOs on rheumatoid arthritis progression. |
| 17 | **Generalizability** | Discuss the generalizability of the study results (a) to other populations, (b) across other exposure periods/timings, and (c) across other levels of exposure | Furthermore, the study used a large sample size and SNPs from GWASs in European population, which provided sufficient statistical validity to estimate causality. Finally, we applied a series of sensitivity analyses to ensure the consistency and the robustness of causal estimates.  First, considering the problem of sample overlap, this study did not find a large sample of rheumatoid arthritis -related GWAS studies in East Asian populations, and APOs-related GWAS studies were limited, making it impossible to draw the same clear conclusions as European populations. |
|  |  |  |  |
| 18 | **Funding** | Describe sources of funding and the role of funders in the present study and, if applicable, sources of funding for the databases and original study or studies on which the present study is based | This work was supported by the Shandong Provincial Medical Association (YXH2022PT06001), the National Key Research and Development Program of China (2021YFF1201101) and ECCM Program of Clinical Research Center of Shandong University (2021SDUCRCE001). |
| 19 | **Data and data sharing** | Provide the data used to perform all analyses or report where and how the data can be accessed, and reference these sources in the article. Provide the statistical code needed to reproduce the results in the article, or report whether the code is publicly accessible and if so, where | This study uses publicly available datasets, which can be found in the IEU Open Project (https://gwas.mrcieu.ac.uk/), and the FinnGen study (https://www.finngen.fi/en). |
| 20 | **Conflicts of Interest** | All authors should declare all potential conflicts of interest | The authors declare that the research was conducted in the absence of any commercial or financial relationships that could be construed as a potential conflict of interest. |

This checklist is copyrighted by the Equator Network under the Creative Commons Attribution 3.0 Unported (CC BY 3.0) lice.

[1] SKRIVANKOVA V W, RICHMOND R C, WOOLF B A R, et al. Strengthening the Reporting of Observational Studies in Epidemiology Using Mendelian Randomization: The STROBE-MR Statement [J]. Jama, 2021, 326(16): 1614-21.

[2] SKRIVANKOVA V W, RICHMOND R C, WOOLF B A R, et al. Strengthening the reporting of observational studies in epidemiology using mendelian randomisation (STROBE-MR): explanation and elaboration [J]. BMJ (Clinical research ed), 2021, 375: n2233.
